# Consensus on the management of united airways disease with type 2 inflammation: a multidisciplinary Delphi study

**DOI:** 10.1186/s13223-023-00780-9

**Published:** 2023-04-23

**Authors:** Marina Blanco-Aparicio, Javier Domínguez-Ortega, Carolina Cisneros, Carlos Colás, Francisco Casas, Alfonso del Cuvillo, Isam Alobid, Santiago Quirce, Joaquim Mullol

**Affiliations:** 1 Department of Respiratory Medicine, Complexo Hospitalario Universitario de A Coruña, A Coruña, Spain; 2 Department of Allergy, La Paz University Hospital, Institute for Health Research (IdiPAZ), CIBER of Respiratory Diseases (CIBERES), Madrid, Spain; 3 Department of Pulmonology, Hospital Universitario La Princesa, Health Research Institute (IP), Madrid, Spain; 4 Department of Allergy, Hospital Clínico-Instituto de Investigación Sanitaria de Aragón, Zaragoza, Spain; 5 Department of Respiratory Medicine, Hospital Universitario Clínico San Cecilio, Granada, Spain; 6 Rhinology & Asthma Unit, ENT Department, Hospital Universitario de Jerez, Cádiz, Spain; 7 Rhinology Unit & Smell Clinic, ENT Department, CIBERES, Hospital Clinic Barcelona, Universitat de Barcelona, C/ Villarroel 170, 08036 Barcelona, Spain

**Keywords:** Asthma, Biologics, Chronic rhinosinusitis with nasal polyps, Type 2 inflammation, United airway disease

## Abstract

**Background:**

Scientific evidence on patients with multimorbid type 2 asthma and chronic rhinosinusitis with nasal polyps (CRSwNP) from a united airways disease (UAD) perspective remains scarce, despite the frequent coexistence of these entities. We aimed to generate expert consensus-based recommendations for the management of UAD patients.

**Methods:**

Using a two-round Delphi method, Spanish expert allergists, pulmonologists and otolaryngologists expressed their agreement on 32 statements (52 items) on a 9-point Likert scale, classified as appropriate (median 7–9), uncertain (4–6) or inappropriate (1–3). Consensus was considered when at least two-thirds of the panel scored within the range containing the median.

**Results:**

A panel of 30 experts reached consensus on the appropriateness of 43 out of the 52 (82.7%) items. The usefulness of certain biomarkers (tissue and peripheral blood eosinophil count, serum total IgE, and fraction of exhaled nitric oxide [FeNO]) in the identification and follow-up of type 2 inflammation, and assessment of the response to biologics, were agreed. Some of these biomarkers were also associated with disease severity and/or recurrence after endoscopic sinus surgery (ESS). Consensus was achieved on treatment strategies related to the prescription of anti-IL-4/IL-13 or anti-IgE agents, concomitant treatment with systemic corticosteroids, and combining or switching to biologics with a different mechanism of action, considering a number of UAD clinical scenarios.

**Conclusion:**

We provide expert-based recommendations to assist in clinical decision-making for the management of patients with multimorbid type 2 asthma and CRSwNP. Specific clinical trials and real-world studies focusing on the single-entity UAD are required to address controversial items.

**Supplementary Information:**

The online version contains supplementary material available at 10.1186/s13223-023-00780-9.

## Background

The concept of united airways disease (UAD) embodies a comprehensive approach to the management of upper and lower respiratory diseases, which are anatomically and immunologically related [1, 2]. Several UAD phenotypes and underlying endotypes have been described, of which the multimorbidity of asthma and chronic rhinosinusitis with nasal polyps (CRSwNP) is one of the most frequent [2]. The term multimorbidity is used to indicate the clustering and co-occurrence of diseases with a common pathological mechanism in an individual where the primary disease is not clear [3]. Clinically, in Caucasian populations, CRSwNP with asthma is characterized by tissue eosinophilia and high local IgE levels [4] Increasing severity of asthma is associated with greater prevalence and severity of CRSwNP [5, 6] while the multimorbidity of CRSwNP and asthma is associated with more severe sinonasal symptoms and worse quality of life [4]. It has been estimated that asthma affects 30–70% of CRSwNP patients [7], and CRSwNP can be found in approximately 30% of asthma patients [6, 8].

Type 2 (T2) inflammation is the most common endotype in both asthma and CRSwNP Caucasian populations [9, 10], and it is characterized by the presence of Th2 and T2 innate lymphoid cells that secrete T2 cytokines (IL-4, IL-5, and IL-13), eosinophilia, and high IgE titers [11]. Accordingly, several biomarkers are used in routine clinical practice to assess T2 inflammation, including blood (≥ 150–300 cells/µl) and tissue (≥ 10 cells/hpf) eosinophil count, total serum IgE (≥ 100 IU/ml), fractional exhaled nitric oxide (FeNO) ≥ 25–30 ppb and/or positive specific IgE [12, 13].

Currently, combined therapeutic strategies for severe asthma and CRSwNP are mainly focused on reducing systemic corticosteroids (SCS) in maintenance and bursts, thereby minimizing asthma exacerbations and worsening of CRSwNP [12, 13], and increasing the use of biologics [14, 15]. In addition, the clinical benefit of endoscopic sinus surgery (ESS) in asthma outcomes has been consistently reported [16–18]. Nevertheless, clinical practice guidelines for the management of asthma and/or CRSwNP patients still do not consider joint management of multimorbidity from the UAD perspective [12, 13, 19].

Overall, scientific evidence on the management of multimorbid patients is scarce because clinical trials and real-world studies mostly evaluate the treatment of asthma or CRSwNP as single entities, or considering each a comorbid condition, rather than UAD. As such, several studies reporting data on biomarkers [20–22], biologics [23–25], SCS [26–28], and ESS [16–18] have been published in recent years. Consequently, the management of UAD patients, which is a common clinical scenario, constitutes a challenge for the healthcare professionals involved, including allergists, pulmonologists, and otolaryngologists (ENT). In this context, we explored experts’ opinions on several controversial items, with the aim of providing consensus-based recommendations for the identification of phenotypes and underlying endotypes, optimal treatments, and strategies to follow up patients with multimorbid T2 asthma and CRSwNP.

## Methods

### Study design

This study was designed based on a modified Delphi method and included a Spanish multidisciplinary board of allergists, pulmonologists, and ENT specialists with clinical expertise in the management of asthma and CRSwNP. A questionnaire was first developed by a scientific committee and participants were then asked to respond to several statements in a two-round online Delphi survey [29].

### Scientific committee and participants

The scientific committee consisted of 3 allergists (JDO, CCo, SQ), 3 pulmonologists (MBA, CCi, FC), and 3 ENT specialists (JM, AC, IA) with extensive expertise in the management of asthma, CRSwNP and their multimorbidity. The committee members were responsible for developing the Delphi questionnaire, reviewing final outcomes, and interpreting the results. An expert panel of 30 members was selected by the scientific committee from the Spanish Society of Allergy and Clinical Immunology (SEAIC) (n = 10), the Spanish Society of Otorhinolaryngology and Head and Neck Surgery (SEORL-CCC) (n = 10), and the Spanish Society of Pulmonology and Thoracic Surgery (SEPAR) (n = 10). Members were contacted by email to participate as panelists in the Delphi survey. Inclusion criteria were: (1) working in clinical practice within the Spanish National Health System, in public or private centers, and (2) at least five years of expertise in the management of patients with asthma and CRSwNP. Participants were identified across several Spanish geographical regions to guarantee appropriate representativeness. Due to a lack of response, two additional experts from SEAIC and one from SEPAR were contacted.

### Questionnaire

The Delphi questionnaire was drafted after a previously published systematic review that summarized recent evidence on the management of UAD (PROSPERO CRD42021262844) [30]. Briefly, a systematic literature search of international databases (PubMed, Web of Science, and Scopus) was conducted for articles published in English and Spanish from January 2015 to July 2021. The search strategy consisted of nine research questions that were defined using the PICO (P, patient; I, intervention; C, comparator; O, outcome) structure. Systematic reviews, clinical trials, *post hoc* studies, clinical practice guidelines, and observational studies reporting data on the management of T2 asthma and CRSwNP were included. In total, 32 publications were selected and assessed using the Critical Appraisal Skills Programme checklists [30].

Based on the studies retrieved and clinicians’ expertise, controversial issues and unmet needs were identified by the scientific committee. A preliminary draft of the questionnaire was developed and subsequently discussed in a virtual meeting. The scientific committee drew conclusions and suggested several recommendations to formulate the statements that could be proposed to the panel. Finally, the Delphi questionnaire was prepared with 32 statements (52 items) that were grouped into three sections: (1) identification of phenotypes (8 statements); (2) treatment (16 statements); and (3) follow-up (8 statements).

### Consensus and data analysis

Consensus was based on the RAND Healthcare Corporation and University of California at Los Angeles (RAND/UCLA) Appropriateness Method [31]. In the first round, participants were asked to rate each statement on a 9-point Likert scale to assess their agreement or disagreement (each statement scored from 1 [totally disagree] to 9 (totally agree) [32]. In the second round, participants re-evaluated the statements for which consensus was not reached after the first round, taking into account their individual vote as well as the median of the panel. Between the two rounds, the scientific committee reviewed controversial items and experts’ comments.Statements were classified as inappropriate, uncertain, or appropriate when a median score of 1–3, 4–6, or 7–9 was calculated, respectively. The mean absolute deviation around the median was used to measure statistical dispersion. Consensus was achieved if at least two-thirds of the panel scored within the range containing the median; otherwise, it was deemed a lack of consensus. Any item without consensus was classified as uncertain regardless of the median value, while controversy was considered when more than one third of individual scores were within the range opposite the one containing the median. Data were analyzed using Excel and the R statistics package version 4.0.1.

## Results

In total, 30 out of the 33 experts participated in both consultation rounds of the Delphi questionnaire (response rate 90.9%) over a two-month period. Participants had a median (range) of 20 (10–35) years of medical expertise and were from nine different Autonomous Communities in Spain (Additional file 1: Table S1). Among the 52 items comprising the questionnaire, consensus on appropriateness was reached in 30 items (57.7%) in the first round. The remaining 22 items were re-assessed in a second round, after which consensus was reached for 13 additional items. As a result of both rounds, agreement on appropriateness was achieved in 43 of 52 items (82.7%) and none of the items were considered inappropriate.

### Identification of phenotypes

The panel strongly agreed that biomarkers associated with T2 inflammation in patients with severe asthma and CRSwNP are peripheral blood and tissue eosinophil count, FeNO, and specific and serum total IgE (median [M]: 8–9) (Table 1). While appropriate, controversy was found among ENT specialists regarding biomarker cut-off values (Additional file 1: Table S2). Experts considered it appropriate to phenotype T2 inflammation using a combination of different biomarkers to improve diagnostic performance (M: 9), despite being insufficient to predict treatment success (M: 8). Of note, the high variability of peripheral blood or tissue eosinophilia was considered a limitation for its usefulness in T2 phenotyping (M: 8).

Consensus was achieved on the association of some biomarkers with increased severity in patients with T2 asthma and CRSwNP, such as elevated FeNO (M: 7) and peripheral blood (M: 8) and tissue eosinophil count (M: 9). In contrast, the association of elevated total serum IgE (M: 8) and positive specific IgE (skin or blood) (M: 5) with severity was overall controversial for all specialties. Experts agreed on the association of tissue eosinophil count with recurrence after ESS (M: 8), whereas controversy was observed for peripheral blood eosinophil count for allergists and ENT specialists. The panel strongly agreed that loss of smell should be assessed in patients with severe asthma and/or CRSwNP (M: 9).

**Table 1:** Results of the Delphi survey Section 1: identification of phenotypes

Number	Statement	Median	Appropriateness	Consensus/ round
1	Biomarkers associated with type 2 inflammation in patients with severe asthma and chronic rhinosinusitis with nasal polyposis (CRSwNP) are:			
1.1	Tissue eosinophil count (sputum, nasal polyp biopsy, bronchial biopsy) (> 10 cells/HPF)	9	Appropriate	Yes/1st
1.2	Elevated fractional exhaled nitric oxide (FeNO) (≥ 25–30 ppb)	8	Appropriate	Yes/2nd
1.3	Positive specific IgE (in serum or by intra-epidermal test or Prick test)	8	Appropriate	Yes/2nd
1.4	Elevated total serum IgE (≥ 100 IU/ml)	8	Appropriate	Yes/2nd
1.5	Elevated peripheral blood eosinophil count (> 250 cells/µl)	8	Appropriate	Yes/1st
2	Cut-off values for the different biomarkers associated with type 2 inflammation are only identified in patients with severe asthma or CRSwNP independently, but not in patients with both diseases.	7	Appropriate	No (controversy)/2nd
3	Phenotyping type 2 asthma and CRSwNP using a combination of different biomarkers improves diagnostic performance compared to using only one of them.	9	Appropriate	Yes/1st
4	Currently available biomarkers are insufficient for complete phenotyping of type 2 inflammation as predictive of treatment success.	8	Appropriate	Yes/1st
5	The high variability of peripheral blood or tissue eosinophilia over time (spontaneously or with treatment, especially with systemic corticosteroids and/or biologics) limits their usefulness in phenotyping patients with suspected type 2 inflammation.	8	Appropriate	Yes/1st
6	The following biomarkers are associated with increased severity in patients with type 2 asthma and CRSwNP:			
6.1	Elevated total serum IgE	8	Appropriate	No (controversy)/2nd
6.2	Elevated FeNO	7	Appropriate	Yes/2nd
6.3	Elevated peripheral blood eosinophil count	9	Appropriate	Yes/2nd
6.4	Positive specific IgE (skin or blood)	5	Uncertain	No (controversy)/2nd
6.5	Elevated tissue eosinophil count	8	Appropriate	Yes/1st
7	The following biomarkers are associated with recurrence after ESS in patients with type 2 severe CRSwNP with or without comorbid asthma:			
7.1	Elevated peripheral blood eosinophil count	7	Appropriate	No (controversy)/2nd
7.2	Highly elevated tissue eosinophil count (> 50 cells/HPF)	8	Appropriate	Yes/1st
8	In any patient with severe asthma and potential sinonasal disease, loss of smell should be assessed.	9	Appropriate	Yes/1st

Physicians rated their agreement with the statements using a 9-point Likert scale (1, totally disagree; 9, totally agree). Statements were classified as inappropriate, uncertain, or appropriate when the median ranged from 1 to 3, 4–6, or 7–9, respectively. Consensus was achieved when at least two-thirds of the panel scored within any of the ranges, otherwise it was deemed as absence of consensus. Controversy was considered when more than one third of individual scores were within the range opposite the one containing the median.

ESS, endoscopic sinus surgery; IU, international units; HPF, high-power field; ppb, parts per billion.

### Treatment

Most experts agreed on the need to evaluate a combination of parameters, including clinical variables and biomarkers, to predict treatment response in patients with severe T2 asthma and CRSwNP (M: 9) (Table 2). They considered that multimorbidity of several respiratory diseases with T2 inflammation is a risk factor for severity and failure of medical or surgical treatment (M: 9). Consensus was achieved on several statements related to the indication for biologics in patients with UAD, such as the use of anti-IL-4/IL-13 or anti-IgE regardless of the presence of allergy (M: 8), although the level of agreement was lower among allergists. Experts agreed that biologics should be prescribed for one disease or the other whenever the criteria are met (M: 8), and should take into account clinical markers of loss of smell and quality of life (M: 9). Moreover, they considered that a history of previous ESS or its contraindication in patients with severe T2 CRSwNP and asthma supports the prescription of biologics (M: 8.5). In patients undergoing ESS, initiation of biologics should not be delayed in the absence of response to appropriate medical-surgical treatment (M: 8). However, the use of biologics before ESS was uncertain and controversial across specialties (Additional file 1: Table S2).

The panel strongly agreed that calculation of the cumulative annual SCS dose should consider the doses administered for both asthma and CRSwNP (M: 9). Regarding the need for short courses of SCS in patients receiving biologics who have not achieved disease control, the experts indicated that it should not be considered as treatment failure if response criteria for the biologics are met (M: 7). The panel agreed that the combination of two biologics with different mechanisms of action may be necessary in patients with UAD who have not achieved symptom control after medical-surgical treatment and use of a biologic (M: 8). However, they also stated that combining biologics is not advisable due to cost-effectiveness and/or lack of safety evidence (M: 8). Accordingly, most experts recommended switching to biologics with a different mechanism of action, or performing ESS if applicable, in UAD patients who do not respond to biologics, even when an improvement in the variables associated with asthma or CRSwNP is observed (M: 7–9). The experts also recommended extending maintenance treatment for at least 6 months before considering withdrawal or switching in UAD patients who show an initial good response (M: 8.5).

**Table 2:** Results of the Delphi survey Section 2: treatment

Number	Statement	Median	Appropriateness	Consensus/ round
1	There is a need to evaluate a combination of parameters including clinical variables and biomarkers to predict treatment response in patients with severe asthma and CRSwNP with underlying type 2 inflammation endotype.	9	Appropriate	Yes/1st
2	The multimorbidity of several respiratory diseases with type 2 inflammation (asthma plus CRSwNP) in a patient is a risk factor for severity and failure of medical or surgical treatment.	9	Appropriate	Yes/1st
3	In patients with severe type 2 CRSwNP and asthma who have an indication for biologics, the use of anti-IL-4/IL-13 or anti-IgE is independent of the presence of allergy.	8	Appropriate	Yes/1st
4	In patients with both diseases, an indication for a biologic should be made whenever it meets the established indication criteria for either severe type 2 CRSwNP or asthma.	8	Appropriate	Yes/1st
5	A history of previous ESS or its contraindication in patients with type 2 CRSwNP (severe) and asthma supports prescription of the biologic.	8.5	Appropriate	Yes/1st
6	In patients with type 2 CRSwNP (severe) and asthma undergoing ESS, the introduction of biologics should not be delayed in the absence of response to appropriate medical-surgical treatment.	8	Appropriate	Yes/1st
7	In patients with type 2 CRSwNP (severe) and asthma, it is preferable to start the biologic before ESS.	5	Uncertain	No (controversy)/2nd
8	The combination of two biologics with different mechanisms of action may be necessary in patients with severe type 2 asthma and CRSwNP who have not achieved symptom control of any of the diseases with appropriate medical-surgical treatment and the use of a single biologic.	8	Appropriate	Yes/1st
9	The need for short courses of systemic corticosteroids (< 2/year) in patients with severe type 2 asthma and CRSwNP who have not achieved control of any of the diseases with biologics should not be considered as failure if response criteria are met in the disease for which it was indicated.	7	Appropriate	Yes/2nd
10	The combination of two biologics for type 2 inflammation in patients with severe asthma and CRSwNP is not advisable due to cost-effectiveness and/or lack of safety evidence, so it is better to switch to another biologic with a different mechanism of action.	8	Appropriate	Yes/2nd
11	Calculation of the cumulative annual systemic corticosteroid dose in a patient with severe type 2 asthma and CRSwNP should take into account the doses of corticosteroids administered for both asthma and CRSwNP.	9	Appropriate	Yes/1st
12	In patients with severe type 2 asthma and CRSwNP with no response to a biologic treatment (indicated for asthma) in the asthma variables, it is advisable to switch to another biologic with a different mechanism of action, even if there is improvement in the variables associated with sinonasal pathology.	8	Appropriate	Yes/2nd
13	In patients with severe type 2 asthma and CRSwNP with no response to a biologic treatment (indicated for CRSwNP) in the CRSwNP variables, it is advisable to switch to another biologic with a different mechanism of action, even if there is improvement in the variables associated with asthma.	7	Appropriate	Yes/2nd
14	In patients with severe type 2 asthma and CRSwNP with no response to biologic therapy in the CRSwNP variables, it is recommended to:			
14.1	Perform ESS even if there is improvement in asthma-associated variables.	8	Appropriate	Yes/1st
14.2	Switch to another biologic.	9	Appropriate	Yes/2nd
15	In patients with severe type 2 asthma and CRSwNP who have a good initial response to a biologic in either condition, it is advisable to extend maintenance treatment for at least 6 months before considering withdrawal or switching to another biologic.	8.5	Appropriate	Yes/1st
16	In patients with type 2 CRSwNP (severe) and asthma, the indication for treatment with biologics should take into account clinical markers of loss of smell (VAS) and quality of life (SNOT-22).	9	Appropriate	Yes/1st

Physicians rated their agreement with the statements using a 9-point Likert scale (1, totally disagree; 9, totally agree). Statements were classified as inappropriate, uncertain, or appropriate when median ranged from 1 to 3, 4–6, or 7–9, respectively. Consensus was achieved when at least two-thirds of the panel scored within any of the ranges, otherwise it was deemed as absence of consensus. Controversy was considered when more than one third of individual scores were within the range opposite the one containing the median.

CRSwNP, chronic rhinosinusitis with nasal polyposis; ESS, endoscopic sinus surgery; SNOT-22, Sino Nasal Outcome Test − 22; VAS, visual analogue scale.

### Follow-up

The panel strongly agreed on biomarkers associated with T2 inflammation that are useful for the follow-up of patients with severe asthma and CRSwNP treated with biologics, including peripheral blood and tissue eosinophil count (M: 8), and FeNO (M: 8), whereas controversy was observed for serum total IgE (M: 7.5) and specific IgE (M: 5) (Table 3). Allergists showed uncertainty on the usefulness of both biomarkers and ENT specialists considered the association with specific IgE as inappropriate (Additional file 1: Table S2). Similar results were retrieved for recommended biomarkers to monitor the response to biologics. Accordingly, experts considered that biomarkers and clinical criteria used when prescribing biologics should be used to monitor the response, if possible (M: 8). In patients with T2 severe asthma and CRSwNP on biologics, the presence of elevated FeNO was considered by the panelists to increase the risk of developing exacerbations (M: 7). In contrast, tissue eosinophil count, anosmia, and quality of life (M: 8) were considered to be associated with a lack of response to ESS, and thus with recurrence of CRSwNP, while the role of peripheral blood eosinophil count was controversial for allergists and ENT specialists. Panelists strongly agreed that, to ensure proper management of these UAD patients, any healthcare professional involved should be conversant with the use of biomarkers and clinical markers for both diseases (M: 9). The minimum and optimal time for assessing response to biologics was agreed at 6 (M: 9) and 12 (M: 8) months, respectively.

**Table 3:** Results of the Delphi survey Section 3: follow-up

Number	Statement	Median	Appropriateness	Consensus/ round
1	Biomarkers associated with type 2 inflammation that are useful for the follow-up of patients with severe asthma and CRSwNP treated with biologics are:			
1.1	Peripheral blood eosinophil count	8	Appropriate	Yes/1st
1.2	Tissue eosinophil count (sputum, nasal polyp biopsy, bronchial biopsy)	8	Appropriate	Yes/1st
1.3	FeNO	8	Appropriate	Yes/1st
1.4	Serum total IgE	7.5	Appropriate	No (controversy)/2nd
1.5	Specific IgE (in serum or by intra-epidermal test or Prick test)	5	Uncertain	No (controversy)/2nd
2	In patients with severe type asthma and CRSwNP, it is recommended to record the following biomarkers to monitor the response to treatment with biologics:			
2.1	Serum total IgE	8	Appropriate	No (controversy)/2nd
2.2	Peripheral blood eosinophil count	8	Appropriate	Yes/1st
2.3	Tissue eosinophil count (in nasal polyps)	8	Appropriate	Yes/2nd
2.4	FeNO	8	Appropriate	Yes/1st
3	The same biomarkers and clinical criteria used at the time of biologic prescription should be used to monitor these patients.	8	Appropriate	Yes/1st
4	In patients with type 2 asthma (severe) and CRSwNP on biologics, the presence of elevated FeNO increases the risk of developing exacerbations.	7	Appropriate	Yes/2nd
5	In patients with severe type 2 asthma and CRSwNP, any professional, regardless of their specialty, must be aware of the results of the different biomarkers and clinical markers of both diseases to ensure correct management.	9	Appropriate	Yes/1st
6	In patients with type 2 CRSwNP (severe) and asthma, the following biomarkers and clinical markers determine the lack of response to ESS and thus recurrence of CRSwNP:			
6.1	Tissue eosinophil count	8	Appropriate	Yes/1st
6.2	Peripheral blood eosinophil count	7	Appropriate	No (controversy)/2nd
6.3	Anosmia (by VAS or smell test)	8	Appropriate	Yes/1st
6.4	Quality of life (SNOT-22)	8	Appropriate	Yes/1st
7	The optimal time for assessing response to a biologic in patients with severe type 2 asthma and CRSwNP is 12 months.	8	Appropriate	Yes/2nd
8	The minimum time for assessing response to a biologic in patients with severe type 2 asthma and CRSwNP is 6 months.	9	Appropriate	Yes/1st

Physicians rated their agreement with the statements using a 9-point Likert scale (1, totally disagree; 9, totally agree). Statements were classified as inappropriate, uncertain, or appropriate when median ranged from 1 to 3, 4–6, or 7–9, respectively. Consensus was achieved when at least two-thirds of the panel scored within any of the ranges, otherwise it was deemed as absence of consensus. Controversy was considered when more than one third of individual scores were within the range opposite the one containing the median.

CRSwNP, chronic rhinosinusitis with nasal polyposis; FeNO, fraction exhaled of nitric oxide; SNOT-22, Sino Nasal Outcome Test − 22; VAS, visual analogue scale.

## **Discussion**

Given the paucity of scientific evidence supporting certain therapeutic strategies for UAD in clinical practice, a multidisciplinary Delphi survey was conducted to elicit expert-based recommendations. To the best of our knowledge, this is the first Delphi study on the management of patients with multimorbid T2 asthma and CRSwNP from a UAD perspective. Outcomes of the questionnaire revealed an overall 83% rate of consensus among allergists, pulmonologists, and ENT specialists, most of whom agreed on the appropriateness of the proposed items. Among these, experts valued the usefulness of several biomarkers to assess and monitor T2 inflammation, as well as their association with disease severity, response to biologics, or recurrence after ESS. Treatment recommendations were made on the prescription of biologics, concomitant treatment with SCS, and combining or switching to other biologics with a different mechanism of action, considering a number of UAD clinical scenarios.

### Identification of phenotypes

The identification of phenotypes and underlying endotypes is currently the best approach to define UAD and predict the patient’s prognosis [1, 4, 5, 33]. While the expert panel agreed on the association of several biomarkers with T2 inflammation, it should be noted that, for some biomarkers (i.e., elevated FeNO and total serum IgE), the available evidence mostly reports data in asthma patients [9, 21, 34]. Nevertheless, as described in a recent real-world study, T2 inflammation can be effectively evaluated in patients with asthma and CRSwNP using FeNO, blood eosinophil count, and total serum IgE [35]. In contrast, controversy was observed among the experts regarding the cut-off values for T2 biomarkers, as these are defined for asthma or CRSwNP in clinical practice guidelines [12, 13]. Therefore, specific studies to determine cut-off values in UAD patients are warranted [36].

The association of disease severity with elevated serum total IgE in UAD patients was controversial, likely due to a lack of scientific evidence. In fact, a study performed in patients with allergic asthma showed high and variable IgE levels [37]. Interestingly, elevated FeNO has been associated with disease severity in asthma patients [38, 39], but also in multimorbid UAD patients [40]. Most panelists expressed uncertainty on the association of positive specific IgE with UAD severity, as expected considering that this biomarker is commonly related to allergic asthma [41].

Although the association of peripheral blood eosinophil count and recurrence after ESS was overall controversial, allergists considered this statement uncertain while pulmonologists and ENT specialists considered it appropriate. In this regard, a recent study has shown that blood eosinophil count combined with asthma history could predict CRSwNP recurrence [42]. Moreover, high tissue eosinophilia has shown good diagnostic accuracy for predicting the likelihood of recurrence of CRSwNP [43].

### Treatment

Given the common pathological characteristics underlying both asthma and CRSwNP, as well as the increased disease burden in multimorbid patients, integral treatment of T2 inflammation in UAD is warranted [4, 33]. While the anti-IgE biologic omalizumab has been traditionally prescribed for allergic asthma, it is currently also indicated as an add-on therapy with intranasal corticosteroids for the treatment of severe CRSwNP. The anti-IL-4/IL-13 agent dupilumab is indicated for the treatment of both severe asthma and CRSwNP. The expert panel consistently considered the use of anti-IL-4/IL-13 or anti-IgE appropriate overall, regardless of the presence of allergy in UAD patients.

The use of biologics before ESS, in the context of severe uncontrolled asthma and CRSwNP, was uncertain and controversial due to the scant evidence [7]. Indeed, randomized clinical trials (RCTs) report data on patients who received biologics either before or after ESS, such as dupilumab [23], omalizumab [25], benralizumab [44], and mepolizumab [24, 45]. As such, some experts recommended ESS before biologics, and others would prescribe these in patients who had multiple interventions and/or contraindication for ESS. It should be noted that the indication for biologics in severe disease has not yet been defined [36].

Experts agreed on the appropriateness of combining two biologics in UAD patients who have not achieved disease control. However, combination of these agents remains controversial, with very limited evidence and only in asthma patients [46]. Since this approach has not been widely explored in RCTs, it is not considered in the indication for currently approved biologics. Although the future of biologics might be focused on different therapeutic targets simultaneously, the high cost of combined therapy and the lack of safety outcomes from real-world evidence could hinder its implementation in clinical practice. Switching to a biologic agent with a different mechanism of action could be a more feasible approach in UAD patients who do not respond to a certain biologic. However, this would depend on the level of improvement and/or worsening in the variables associated with asthma and CRSwNP. To date, the effectiveness of switching between biologics has been only reported in severe asthma patients [47–49].

### Follow-up

For the follow-up of UAD patients receiving biologics, experts agreed on the appropriateness of T2 biomarkers such as peripheral blood and tissue eosinophil count, in line with those established for the indication of the treatment [12, 13, 50]. In contrast, the use of total serum IgE was controversial because this biomarker has not been proven to be associated with a response to biologics. While dupilumab and omalizumab have been shown to reduce circulating IgE [36], its serum level can vary during treatment. The expert panel considered that the best approach would be a combination of biomarkers to ensure more comprehensive follow-up of UAD patients. Since biomarkers predicting response to biologics in CRSwNP have not yet been described in the literature, the need for further research was stressed.

FeNO has been associated with asthma exacerbations and lung function [39]. In patients with asthma and CRSwNP treated with certain biologics such as dupilumab or tezepelumab, FeNO has been identified as a biomarker that can predict the response to therapy [20, 22]. While the use of tissue eosinophil count as a biomarker for ESS is broadly agreed, the role of peripheral blood eosinophil count in this context remains controversial. Plaza et al. evaluated the impact of bilateral functional ESS, and found a trend towards a lower serum eosinophil count in patients with asthma and severe CRSwNP [16]. Furthermore, several studies have described the impact of ESS in UAD patients, not only related to the improvement in CRSwNP outcomes, but also on respiratory function and asthma control [17, 18, 51].

Among the clinical markers that the expert panel considered appropriate to determine a lack of response to ESS, anosmia is not currently used in clinical practice. Accordingly, although there was overall agreement, differences were observed among specialties. Recent studies have demonstrated poor mid- to long-term efficacy of ESS in the loss of smell, apart from the effect of initial surgeries [52, 53]. On the other hand, a consensus was reached on the appropriateness of minimal and optimal time for assessing response to biologics, in line with a recent real-world study [15].

This study is subject to certain limitations, which are mainly related to the Delphi methodology. To minimize imposed preconceptions and ensure impartiality in the design of the questionnaire, all statements were thoroughly reviewed and subsequently agreed upon by the scientific committee members. It is worth noting that the experts’ evaluation may be affected by personal interpretation of the statements and/or their own clinical expertise. Considering that there might be a potential bias in the selection of the expert panel, a large number of specialists from different Autonomous Communities in Spain were included.

## Conclusion

In summary, this Delphi survey provides expert-based recommendations that may assist healthcare professionals involved in the management of multimorbid T2 asthma and CRSwNP patients, and guide clinical decision-making from the UAD perspective. Although the level of consensus among specialists constituting the expert panel was overall high, some controversies came to light. Therefore, we emphasize the need for specific clinical trials and real-world studies considering the single-entity UAD to further support consensus recommendations, but also to address the unmet needs identified herein. Future research will help to ascertain the best management algorithm and therapeutic strategies according to the patient profile and history, and eventually enable evidence-based implementation of clinical practice guidelines.

## Supplementary Information

**Additional file 1: Table S1.** Characteristics of the study expert participants.**Table S2.** Data on controversial items in the Delphi survey by specialty.

## Data Availability

All data relevant to the study are included in the article or uploaded as supplementary information.

## Abbreviations

CRSwNP: Chronic rhinosinusitis with nasal polyps
ENT: Otolaryngologists
ESS: Endoscopic sinus surgery
FeNO: Fraction of exhaled nitric oxide
SCS: Systemic corticosteroids
T2: Type 2
UAD: United airways disease
