# Supplementary material for: Consensus on the management of united airways disease with type 2 inflammation: a multidisciplinary Delphi study
Source: Allergy Asthma Clin Immunol. 2023 Apr 23;19:34. doi: 10.1186/s13223-023-00780-9 (PMC10124060; doi:10.1186/s13223-023-00780-9)
Supplement: Supplementary file 1 — Additional file 1: Table S1. Characteristics of the study expert participants.Table S2. Data on controversial items in the Delphi survey by specialty. [file 13223_2023_780_MOESM1_ESM.docx]

**Additional material**

**Table S1.** Characteristics of the study expert participants.

| **Characteristics** | **Total (n = 30)** |
| --- | --- |
| Years of experience, median [range] | 20 [10 – 35] |
| Specialty, n (%)  Allergy  Pulmonology  Otolaryngology (ENT) | 10 (33.3)  10 (33.3)  10 (33.3) |
| Spanish Autonomous Communities, n (%)  Madrid  Andalusia  Catalonia  Galicia  Valencia  Navarre  Murcia  Aragon  Canary Islands | 8 (26.7)  8 (26.7)  6 (20.0)  2 (6.7)  2 (6.7)  1 (3.3)  1 (3.3)  1 (3.3)  1 (3.3) |

**Table S2**. Data on controversial items in the Delphi survey by specialty.

| **Number** | **Statement** | **ALLERGY**  **(n=10)** | | **PULMONOLOGY**  **(n=10)** | | **ENT Specialists**  **(n=10)** | |
| --- | --- | --- | --- | --- | --- | --- | --- |
|  |  | **Median** | **Appropriateness/ consensus** | **Median** | **Appropriateness/ consensus** | **Median** | **Appropriateness/ consensus** |
| **SECTION 1. IDENTIFICATION OF PHENOTYPES** | | | | | | | |
| 2 | Cut-off values for the different biomarkers associated with type 2 inflammation are only identified in patients with severe asthma or CRSwNP independently, but not in patients with both diseases. | 7 | Appropriate / yes | 7.5 | Appropriate / yes | 7.5 | Appropriate / no (controversy) |
| 6 | The following biomarkers are associated with increased severity in patients with type 2 asthma and CRSwNP: | | | | | | |
| 6.1 | Elevated serum total IgE | 7.5 | Appropriate / no (controversy) | 8 | Appropriate / no (controversy) | 9 | Appropriate / no (controversy) |
| 6.4 | Positive specific IgE | 5 | Uncertain / no (controversy) | 5 | Uncertain / no (controversy) | 7 | Appropriate / no (controversy) |
| 7 | The following biomarkers are associated with recurrence after ESS in patients with type 2 CRSwNP with or without comorbid asthma: | | | | | | |
| 7.1 | Elevated peripheral blood eosinophil count | 6 | Uncertain / no (controversy) | 8 | Appropriate / yes | 7.5 | Appropriate / no (controversy) |
| **SECTION 2. TREATMENT** | | | | | | | |
| 7 | In patients with type 2 CRSwNP (severe) and asthma, it is preferable to start the biologic before ESS. | 5 | Uncertain / yes | 5 | Uncertain / yes | 4.5 | Uncertain / no (controversy) |
| **SECTION 3. FOLLOW-UP** | | | | | | | |
| 1 | Biomarkers associated with type 2 inflammation that are useful for the follow-up of patients with severe asthma and CRSwNP treated with biologics are: | | | | | | |
| 1.4 | Elevated serum total IgE | 5 | Uncertain / no (controversy) | 8.5 | Appropriate / yes | 8.5 | Appropriate / no (controversy) |
| 1.5 | Positive specific IgE | 4 | Uncertain / no (controversy) | 8 | Appropriate / no (controversy) | 3 | Inappropriate / no (controversy) |
| 2 | In patients with severe type asthma and CRSwNP, it is advisable to record the following biomarkers to monitor the response to treatment with biologics: | | | | | | |
| 2.1 | Elevated serum total IgE | 6.5 | Appropriate / no (controversy) | 8.5 | Appropriate / no (controversy) | 9 | Appropriate / yes |
| 6 | In patients with type 2 CRSwNP (severe) and asthma, the following biomarkers and clinical markers determine the lack of response to ESS and thus recurrence of CRSwNP: | | | | | | |
| 6.2 | Elevated peripheral blood eosinophil count | 5.5 | Uncertain / no (controversy) | 7 | Appropriate / yes | 8.5 | Appropriate / no (controversy) |

CRSwNP, chronic rhinosinusitis with nasal polyposis; ENT, ear, nose and throat; ESS, endoscopic sinus surgery.
